# Obesity and cholesterol: A cross-sectional analysis of Qatar Biobank data

**DOI:** 10.5339/qmj.2025.97

**Published:** 2025-12-01

**Authors:** Nada Arar, Nasra Ayon, Rodaina Hashem, Nour Alhussaini, Manar E. Abdel-Rahman

**Affiliations:** 1 Department of Public Health, College of Health Sciences, QU Health, Qatar University, Doha, Qatar *Email: melhassan@qu.edu.qa

**Keywords:** Total cholesterol, low-density lipoprotein, high-density lipoprotein, obesity, biobank, Qatar

## Abstract

**Background:**

Cardiovascular disease (CVD) remains a major health concern in Qatar, with ischemic heart disease being the leading cause of mortality. Suboptimal cholesterol levels represent a major risk factor for CVD. Suboptimal cholesterol levels are highly prevalent among adults worldwide, including in the Middle East and North Africa region. Obesity, a major risk factor for suboptimal cholesterol, is highly prevalent in Qatar and contributes significantly to both mortality and morbidity.

**Objective:**

To estimate the prevalence of total cholesterol (TC), low-density lipoprotein (LDL), and low high-density lipoprotein (HDL) levels in a specific population in Qatar, and to assess their associations with obesity.

**Methods:**

Data for this cross-sectional study was derived from the Qatar Biobank, a population-based biobank that recruits Qatari nationals or long-term residents aged 18 years and above. Multivariable logistic and linear regression models were used to assess the associations between TC, LDL, HDL, and obesity levels defined by body mass index (BMI) and waist circumference (WC).

**Results:**

Of 1,000 participants, 920 were included in the study. Approximately 37% of women had higher TC (≥ 5.18 mmol/L), 28% had higher LDL (≥ 3.4 mmol/L), and 36% had lower HDL (<1.3 mmol/L). Among men, 43% had higher TC, 41% had higher LDL, and 22% had lower HDL. HDL levels were significantly associated with higher BMI and WC in both women (adjusted odds ratio [aOR] for obese vs normal weight = 3.60, 95% CI: 1.86–6.94; aOR for higher WC vs lower WC = 2.35, 95% CI: 1.42–3.90) and men (aOR for obese vs normal weight = 3.00, 95% CI: 1.48–6.11; aOR for higher WC vs lower WC = 2.12, 95% CI: 1.27–3.55). In men, higher BMI was significantly associated with increased odds of higher TC (aOR = 2.45, 95% CI: 1.38–4.36) and LDL (aOR = 2.56, 95% CI: 1.42–4.56), while no significant associations were found in women.

**Conclusion:**

Both women and men with higher BMI and WC had significantly lower HDL levels. Additionally, men with higher BMI were at increased risk for higher TC and LDL levels, whereas no significant associations were observed in women. Further research is needed to examine the associations between cholesterol types and metabolic syndrome components in representative populations with adequate sample sizes.

## 1. BACKGROUND

Cardiovascular disease (CVD) is the leading cause of mortality, disability-adjusted life years, and economic burden in Qatar,^1^ with ischemic heart disease being the primary cause of death.^2^ Higher levels of total cholesterol (TC) and low-density lipoprotein (LDL), along with lower levels of high-density lipoprotein (HDL), are key risk factors for CVD.^3,4^ However, cardiovascular risk prediction models, such as the Framingham Risk Score and SCORE2, highlight non-HDL cholesterol as a more comprehensive marker of cardiovascular risk.^5,6^ Studies have shown that lower TC and LDL levels, along with higher HDL levels, can reduce the global incidence of CVD.^7,8^

Among the Qatari population, high LDL is one of the leading causes of death, with the percentage of all-age deaths attributed to it increasing from 0.093 to 0.132 between 2010 and 2019.^2^ The lack of studies on potential risk factors associated with optimal cholesterol levels in Qatar highlights the need for further research in this area to guide effective public health interventions and policies.

Evidence suggests that several factors—including diet, physical activity, tobacco use, family history, age, gender, and other medical conditions—contribute to suboptimal cholesterol levels.^9–13^ and directly influence the risk of CVD.^14^ Obesity, a major global public health problem, is a significant risk factor for suboptimal cholesterol levels due to its effects on lipid and cholesterol metabolism.^12,15–18^ Additionally, studies have shown that the combined effect of genetic predisposition and obesity can influence HDL cholesterol levels in individuals.^19^

In Qatar, obesity rates increased by 110.2% between 2007 and 2017, posing a significant risk factor for both mortality and morbidity.^20^ A recent study reported obesity prevalence of 46% among Qatari students and 41% among non-Qatari students;^21^ in another study, this prevalence ranged from 34.7% to 37.6% across the three regions of Qatar. ^21^ The 2012 Qatar STEPwise survey indicated that 41% of adults in the country were classified as obese.^22^

Despite the high prevalence of obesity in Qatar, there is a paucity of studies examining its association with cholesterol levels in the population. A study conducted in 2012 in Qatar found significant associations between cholesterol levels and waist circumference (WC) among school children.^23^ The Middle East and North Africa (MENA) region is considered high-risk for metabolic disorders due to its cultural, historical, and socioeconomic diversity, which contributes to varying health statuses, inadequate healthcare management, high levels of physical inactivity, poor diet, and insufficient prevention programs.^24^ Given the significant role of cholesterol in the development of ischemic heart disease, understanding obesity as a risk factor is essential for designing effective prevention and intervention strategies. This study aims to fill this knowledge gap in Qatar’s research database and contribute to reducing the incidence of cholesterol-related diseases. The Qatar Biobank (QBB), currently under the Qatar Precision Health Institute, presents a unique opportunity to investigate this association by providing access to data from a large population.^25^

The aim of this study is to estimate the prevalence of cholesterol levels among the QBB population and assess their association with obesity, using body mass index (BMI) and WC as a more accurate measure of central adiposity.^26^ Specifically, the study aims to answer the following research questions: What are the levels of TC, LDL, and HDL, and how are they related to obesity levels within the QBB population? We hypothesize that obesity is a determinant of suboptimal cholesterol levels.

## 2. METHODS

### 2.1. Data sources

Data for this study was obtained from the QBB, a center operating under the umbrella of the Qatar Foundation,^27^ and one of the largest population-based biobanks in the Gulf Cooperation Council countries. Since its launch in 2012, QBB has aimed to recruit 60,000 participants and follow them every five years, collecting data on genetic, metabolic, health, lifestyle, and environmental risk factors for diseases. Detailed QBB methodologies have been published elsewhere.^25^

### 2.2. Study design and sample size

This cross-sectional study uses secondary baseline data from the QBB population-based cohort. The study includes data from volunteers who are either Qatari nationals or long-term residents of Qatar (living in the country for 15 years or more), aged 18 years or older, regardless of gender. The study sample was drawn from the QBB population-based cohort and consisted of 1,000 randomly selected participants. Of these, 80 participants were excluded from the study due to missing data on the main exposure or study outcome, resulting in a final sample of 920 participants.

To ensure transparent and comprehensive reporting, the study followed the guidelines outlined in the Strengthening the Reporting of observational studies in epidemiology (STROBE) statement.

### 2.3. Study variables

The main outcome variables in this study are TC, LDL, and HDL levels, measured in millimoles per liter (mmol/L). These variables were analyzed both as continuous measures and as categorical variables, classified into lower and higher levels based on the following cutoff points: TC: <5.18 or ≥5.18 mmol/L; LDL: <3.4 or ≥3.4 mmol/L; HDL for women: <1.3 or ≥1.3 mmol/L; and HDL for men: <1.0 or ≥1.0 mmol/L.^28^

The main exposure variable is the level of obesity, assessed using participants’ BMI and WC and analyzed as both continuous and categorical variables. BMI was calculated as weight [in kilogram (kg)] divided by height squared [in meters (m)]. Based on World Health Organization cut-offs, participants with a BMI of 18.5–24.9 kg/ m^2^ were classified as normal weight, 25.0–29.9 kg/ m^2^ as overweight, and ≥30.0 kg/m^2^ as obese. Using Qatar-specific cut-offs, male and female participants with WC ≥94 cm and ≥102 cm, respectively, were classified as having higher WC.^29^

The selection of socio-demographic characteristics and other covariates was based on the existing literature^9–13,15,30^ and the availability of data from the QBB.^25^ The variables considered included nationality, gender, age, monthly income (in Qatari Riyal), house ownership, level of education, and current employment status. In addition, information on covariates such as physical activity, sleep patterns, smoking, consumption of fast food and red meat, comorbidities, and personal and family history of diseases was obtained from QBB measurements.^25^

### 2.4. Statistical analysis

Descriptive statistics, including means, standard deviations, and percentages, were used to summarize the characteristics of the participants as well as the levels of TC, LDL, and HDL. Bivariate associations between obesity variables and TC, LDL, and HDL were assessed using Pearson correlations for continuous variables and Chi-square tests for binary variables. Crude and multivariable logistic and linear regression models were employed to assess the potential associations between obesity indicators and levels of TC, LDL, and HDL, respectively. A principled method based on causal assumptions was used to identify confounders, with the set of confounders determined through Directed Acyclic Graph (DAG) analysis,^31^ ensuring consistency and interpretability across models. As previous studies reported gender differences in the association between weight and cholesterol levels,^32^ this study stratified the data analysis by gender. Statistical significance was set at p < 0.05. All analyses were performed using Stata version 18.5.^33^

### 2.5. Ethics approval and consent to participate

Data collection involving human participants in this study was conducted in accordance with the ethical guidelines and regulations outlined in the Declaration of Helsinki. All participants in this study provided informed consent, which was obtained by QBB. The study was approved by the Institutional Review Board at QBB and the relevant authorities in Qatar. To ensure participant confidentiality and prevent potential misuse of information, the data provided by QBB for this study was de-identified. The data will remain de-identified even after the completion of the study.

## 3. RESULTS

### 3.1. Basic characteristics

Of the 1000 participants originally enrolled in QBB, 920 were included in this study. Eighty participants were excluded due to missing data for TC, LDL, or HDL (3%) or BMI (7.7%). Participants had a mean age of 40.3 years (SD = 13.1), with a balanced gender distribution. The majority were of Qatari nationality (81%), employed (60%), and held a university degree or higher (40%) (Table 1).

### 3.2. TC, LDL, or HDL levels

**Figure 1:**
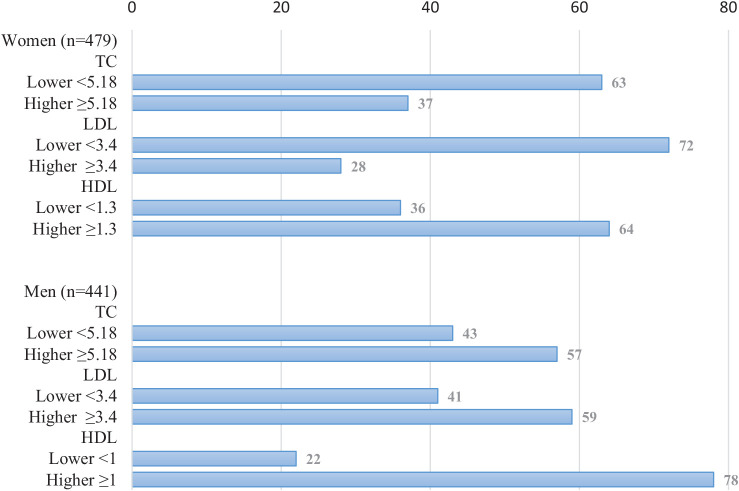
Gender-specific percentages of total cholesterol (TC), low-density lipoprotein (LDL), and high-density lipoprotein (HDL) levels in millimoles per liter (mmol/L) (*n* = 920).

All cholesterol levels were measured in mmol/L. For women, the mean TC was 4.97 (SD = 0.89), the mean LDL was 3.04 (SD = 0.78), and the mean HDL was 1.40 (SD = 0.32). For men, the mean TC was 5.06 (SD = 1.00), the mean LDL was 3.28 (SD = 0.89), and the mean HDL was 1.13 (SD = 0.24). Approximately 40% and 35% of the study populations had lower TC and LDL, respectively. Figure 1 shows that 37% of women had higher TC (≥ 5.18 mmol/L), 28% had higher LDL (≥ 3.4 mmol/L), and 36% had lower HDL (<1.3 mmol/L). Among men, 43% had higher TC (≥5.18 mmol/L), 41% had higher LDL (≥3.4 mmol/L), and 22% had lower HDL (<1.0 mmol/L). Overall, a higher percentage of men had higher TC and LDL, as well as lower HDL levels, compared to women.

### 3.3. Bivariate analysis

**Figure 2:**
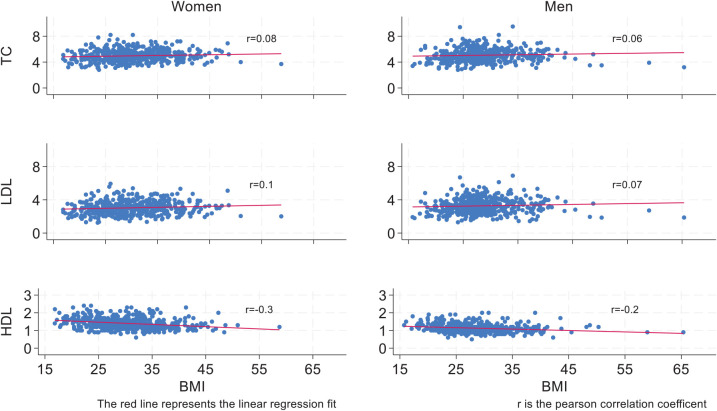
Gender-specific scatter plots of total cholesterol (TC), low-density lipoprotein (LDL), and high-density lipoprotein (HDL) levels in millimoles per liter (mmol/L) by body mass index (BMI) (*n* = 920). The red line represents the linear regression fit for each plot, and r is the Pearson correlation coefficient.

**Figure 3:**
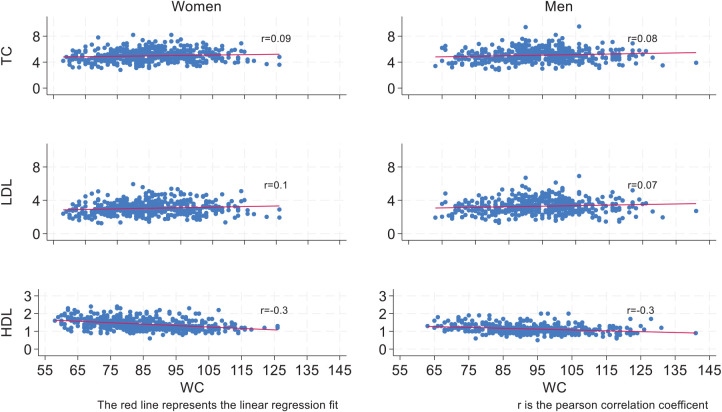
Gender-specific scatter plots of total cholesterol (TC), low-density lipoprotein (LDL), and high-density lipoprotein (HDL) levels in millimoles per liter (mmol/L) by waist circumference (WC) (*n* = 920). The red line represents the linear regression fit for each plot, and r is the Pearson correlation coefficient.

Scatter plots of cholesterol levels in relation to BMI and WC are presented in Figures 2 and 3. For both women and men, the figures generally show a slight increase in TC and LDL levels, and a decrease in HDL levels, as BMI or WC increases.

Table 2 presents the bivariate associations of TC, LDL, and HDL with BMI status and WC, stratified by sex. Approximately 50% of women and 35% of men were obese, and approximately 27% of both women and men had higher WC. Our findings indicate that higher TC levels were significantly more prevalent among overweight (49%) and obese men (46%) compared to men with normal BMI (29%) (*p* = 0.001). However, this association was not observed in women (*p* = 0.390). Similar results for LDL were observed in women (*p* = 0.508) and men (*p* = 0.001). There was no significant association between TC and WC or between LDL and WC in either men or women. Conversely, HDL showed a statistically significant association with both BMI status and WC in both women and men. Lower HDL levels were more prevalent among overweight (45%) and obese women (34%) compared to women with normal BMI (20%)

(p < 0.001). These percentages were lower among men (28% vs 23% vs 14%, *p* = 0.038). Similarly, both women and men with higher WC were more likely to have low HDL levels (53% higher vs 30% normal, p < 0.001 in women; and 31.9% vs 18.9%, *p* = 0.004 in men).(*n* = 920).

The bivariate analysis among women identified that age, education, sleep, water pipe (shisha) smoking, history of high cholesterol, mother’s history of heart disease, and mother’s history of overweight/obesity were significantly associated with TC levels. Among men, these factors included income and a history of high cholesterol (see Supplementary Tables 1 and 2). LDL levels were significantly associated with age, sleep, and history of high cholesterol in women, and with income, smoking, and history of high cholesterol in men. Lastly, HDL levels were significantly associated with employment, physical activity, sleep, history of diabetes, and treatment for cholesterol in women, and with house ownership in men (see Supplementary Tables 1 and 2).

### 3.4. Adjusted analysis

Table 3 presents gender-stratified unadjusted and adjusted models used for assessing the associations between BMI status and higher levels of TC, LDL levels, as well as lower HDL levels. Results revealed a strong and significant positive association between higher BMI status and lower HDL levels among women (adjusted odds ratio [aOR] for overweight vs normal weight = 2.23, 95% confidence interval [CI]: 1.14 – 4.36; aOR for obese vs normal weight = 3.38, 95% CI: 1.76–6.49). Similar associations were observed among men (aOR for overweight vs normal weight = 2.33, 95% CI: 1.16–4.70; aOR for obese vs normal weight = 2.81, 95% CI: 1.39–5.71). Similar results were observed when BMI was treated as a continuous variable where for every 10-unit increase in BMI, the odds of having lower HDL levels decreased by 13% in women (aOR: −0.13, 95% CI: −0.19 to −0.08). In men, this decrease was 5% (aOR: 0.05, 95% CI: −0.06 to −0.03).

Among men, being overweight or obese was significantly associated with increased risks of higher TC and LDL levels. Compared to men with normal weight, those who were overweight were more than twice as likely to have higher TC (aOR = 2.59, 95% CI: 1.49–4.50) and higher LDL (aOR = 2.59, 95% CI: 1.48–4.54). Obese men showed similarly elevated risks of high TC and LDL levels (TC: aOR = 2.27, 95% CI: 1.28 – 4.02; LDL: aOR = 2.76, 95% CI: 1.55–4.93). In contrast, the positive but non-significant associations between obesity and elevated TC and LDL levels in the crude models for women weakened after adjustment; the adjusted results remained non-significant showing minimal to no effect.

In both women and men, higher WC was significantly associated with lower HDL levels, with aORs indicating increased odds of lower HDL levels in women (aOR = 2.35, 95% CI: 1.42–3.90) and men (aOR = 2.12, 95% CI: 1.27–3.55) (Table 4). For continuous WC, each 10-unit increase was associated with a reduction in HDL levels (women: adjusted *β* = −0.13, 95% CI: −0.19 to −0.08; men: adjusted *β* = −0.08, 95% CI: −0.12 to −0.04). However, after adjustment, no significant associations were observed between either categorical or continuous WC and higher TC or LDL levels in either sex.

Overall, the results suggest that while higher BMI was significantly associated with HDL levels in both women and men, men exhibited more pronounced associations with TC and LDL levels than women. In contrast, higher WC was significantly associated with lower HDL levels in both sexes, but showed no significant association with TC or LDL levels.

## 4. DISCUSSION

Obesity is a major public health concern that continues to increase the burden of morbidity and mortality. It is a complex, multifactorial condition resulting from the interaction of genetic, environmental, and behavioral factors. Obesity is associated with a variety of health complications, including suboptimal cholesterol levels, which further exacerbate the risk of CVD and other chronic illnesses.^3,4,7^

This study found a significant positive association between obesity and lower HDL levels in both women and men. Lower HDL levels were observed in overweight and obese individuals compared to those with normal weight, and in participants with higher WC compared to those with lower WC. Other studies have also shown an inverse correlation with HDL, indicating that as BMI increases, HDL decreases.^34–36^ For example, a large cohort study involving diverse populations found an inverse correlation, reinforcing the consistent finding across various research designs.^36^ Moreover, weight loss studies have shown that reductions in body fat can improve HDL levels, whereas high-fat diets can blunt these improvements, suggesting that dietary factors also play a significant role.^34^ HDL cholesterol levels remained stable despite weight regain following bariatric surgery, according to a five-year retrospective, observational cohort study.^35^ Another study demonstrated that individuals with higher WC exhibit a significant increase in the proportion of smaller HDL3c particles—which are considered less cardioprotective—compared to those with lower WC. This shift in HDL subfraction distribution may contribute to the reduced HDL-C levels observed in individuals with central obesity.^37^

Our cross-sectional study of the QBB population revealed gender-specific differences in the magnitude of the association between obesity status and levels of TC, LDL, and HDL. Among men, obesity status was strongly and significantly associated with higher levels of TC and LDL after adjusting for covariates. However, for women, the associations were positive initially but reversed and became non-significant or minimal after adjusting for covariates. Consistent with our findings, several studies have shown that overweight and obese men are more likely to have higher cholesterol levels than women.^34,38^ This could be due to the estrogen hormone, which plays a significant role as a preventive factor against acquiring high cholesterol in women,^39^ and genetic factors.^40^ However, it is important to note that these findings are not consistent across all studies, as some have reported no gender differences.^41^

Previous research has found a clear association between obesity levels and an increased risk of elevated TC, LDL, and other CVD risk factors. Consistent with our findings in men, a study from England found that higher TC was more common in the morbidly obese group (men: 48.2%, women: 36.3%) compared to those with normal weight (men: 25.0%, women: 20.0%).^12^ Similarly, ORs for higher LDL increased from 1.58 (95% CI: 1.22–2.05) among participants with a BMI of 22–23 kg/m^2^ to 4.8 (95% CI: 2.84–8.31) among those with a BMI ≥ 30 kg/m^2^ in a Korean population.^36^ These findings are consistent with other studies that have reported a positive association between BMI and cholesterol levels.^38,42–45^ This may be attributed to unhealthy dietary habits, including high consumption of saturated fatty acids and cholesterol-rich foods.^46^ However, the exact mechanisms relating obesity to high TC levels remain uncertain. One proposed explanation is that prolonged weight gain impairs renal nephron dysfunction, resulting in increased arterial pressure.^47^

Our study found that approximately 40% of the participants had high TC levels (≥5.2 mmol/L), with a higher prevalence in men (43.1%) than in women (37%). This estimate is consistent with the global prevalence of high TC in adults (39%) and the 38% prevalence reported in the MENA region, which includes Qatar.^7^ According to the Qatar Ministry of Public Health (MoPH) STEPwise survey conducted in 2012, the prevalence of raised TC (TC ≥ 5.0 mmol/L or currently taking medication for raised cholesterol) was 21.9%.^22^ In this survey, the prevalence of LDL ≥ 3.4 mmol/L was 9%, while the prevalence of HDL < 1.3 mmol/L was 37.3% in females and 49.2% in males. Our findings suggest that the prevalence of higher levels of TC and LDL, along with lower levels of HDL, among QBB participants are much higher than the prevalence reported by the MoPH in 2012. Although our study and the STEPwise survey were conducted using different methods and populations at different times, the significant difference in prevalence rates between the two studies highlights growing public health concerns regarding suboptimal cholesterol levels in Qatar. It is worth noting that, although our study population may have been healthier than the general population due to self-selection bias, they still exhibited suboptimal cholesterol levels. This suggests that the issue is prevalent even among a relatively healthier population. Therefore, addressing this issue through lifestyle modifications and medical interventions should be prioritized in Qatar.

While our study provides important insights into TC, LDL, and HDL levels in Qatar, it is worth noting that healthcare professionals also assess cholesterol ratios, such as the LDL-to-HDL cholesterol ratio. This is because higher levels of LDL cholesterol and/or an elevated LDL-to-HDL ratio can increase the risk of developing CVD, even if TC levels are within the normal range. Therefore, while our study addresses an important aspect of the problem, future research should investigate additional cholesterol markers to achieve a more comprehensive understanding of the cardiovascular health in Qatar’s population.

## 5. LIMITATIONS AND STRENGTHS

This study has several limitations that should be considered. First, as this is a cross-sectional study, casual relationships between the variables cannot be established. Nonetheless, several key prevalence data and gender-specific associations between BMI and cholesterol provide a foundation for future longitudinal studies to explore causal relationships in this population. Second, the relatively small sample size—constrained by QBB data access limitations—may have reduced the statistical power needed to detect a significant association between cholesterol levels and obesity. While a larger sample could increase statistical power, this subset still represents a diverse cross-section of adults in Qatar and provides meaningful insights into the association under study. Third limitation is that the study population consisted primarily of volunteers, which may have introduced selection bias. There is a risk that volunteers may be more health-conscious or better educated, which could limit the generalizability of our findings to the broader Qatari population. Finally, because the study relied on self-reported data, there is a potential risk of recall bias affecting the accuracy of the data.

Despite these limitations, our study has several strengths. To our knowledge, this is the first study to investigate the association between obesity and cholesterol levels in Qatar. We adjusted for several potential covariates in our analysis, as identified in the literature. This allowed us to achieve a more accurate assessment of the association between obesity and cholesterol levels in the QBB population.

While the association between obesity and CVD has been widely documented in global research,^48^ this study provides unique insights by focusing on the Qatari population. Qatar’s unique demographic and lifestyle factors provide an important context for understanding these health relationships. The MENA region, including Qatar, experiences a high prevalence of CVD risk factors, notably dyslipidemia, obesity, and diabetes.^7,24,49,50^ These conditions are influenced by lifestyle patterns, including high-calorie diets and sedentary habits, which are prevalent in this region^51,52^ and may differ from global patterns. Our findings add important data to an understudied population within the MENA region, where relatively few population-specific studies have assessed the complex relationships between obesity and cholesterol.

## 6. CONCLUSION

In conclusion, our study investigated the association between obesity and cholesterol, stratified by gender. Our findings indicate that a larger proportion of men with higher TC and LDL levels and lower HDL levels were overweight or obese compared to those with normal BMI. In women, the association was observed only for HDL. However, larger studies with representative populations and sufficient sample sizes are needed to further investigate associations between different types of blood cholesterol and other components of metabolic syndrome.

Given the high prevalence of obesity in Qatar, effective public health interventions are essential. Health promotion programs can increase awareness about the risk factors for obesity and their potential health outcomes. Furthermore, interventions aimed at reducing obesity—such as promoting physical activity and healthy eating habits—may help reduce the burden of suboptimal cholesterol levels and associated health complications in Qatar.

## LIST OF ABBREVIATIONS

**Table:** 

aOR	Adjusted Odds Ratio
BMI	Body Mass Index
CVD	Cardiovascular Disease
HDL	High-Density Lipoprotein
LDL	Low-Density Lipoprotein
MENA	Middle East and North Africa
mmol/L	Millimoles per Liter
MoPH	Qatar Ministry of Public Health
QBB	Qatar Biobank
TC	Total Cholesterol
WC	Waist Circumference

## ETHICS APPROVAL AND CONSENT TO PARTICIPATE

The Institutional Review Board (IRB) at QBB approved the provision of data for this study in compliance with ethical principles (IRB protocol number: Ex-2018-RES-ACC-0119-0061). Additionally, ethical approval was obtained from the Qatar University Institutional Review Board (approval number: QU-IRB 1256-E/20).

## AVAILABILITY OF DATA AND MATERIALS

The dataset generated for this study is available upon request from the Qatar Biobank Study data management team. In accordance with Qatar Biobank Institutional Review Board approval (protocol number: Ex-2018-RESACC-0119-0061) and the terms of the Data and Material Transfer Agreement, we are not permitted to share the data independently, without prior written consent from the provider.

## AUTHORS’ CONTRIBUTIONS

NA, NA, RH, and NWZ: Formal analysis, writing—original draft. MEA: Conceptualization, data curation, formal analysis, supervision, methodology, writing—review & editing. All authors reviewed and approved the final manuscript.

## ACKNOWLEDGMENTS

The authors would like to thank Qatar Biobank for their support in providing the data used to establish this project.

## Figures and Tables

**Table 1.** Socio-demographic characteristics of participants (*n* = 920).

Age in years, Mean [Standard deviation]	40.3 [13.1]
**Age categories in years, *n* (%)**	
18–29	228 (24.8)
30–39	254 (27.6)
40–49	208 (22.6)
50+	230 (25.0)
**Gender, *n* (%)**	
Women	479 (52.1)
Men	441 (47.9)
**Nationality, *n* (%)**	
Non-Qatari	175 (19.0)
Qatari	745 (81.0)
Highest level of education*, *n* (%)	
Primary or below	122 (13.3)
Secondary	420 (45.7)
University+	367 (39.9)
Own house*, *n* (%)	
No	278 (30.2)
Yes	631 (68.6)
Total monthly income in QR**, *n* (%)	
0–10,000	225 (24.5)
10,001–20,000	234 (25.4)
20,001–50,000	287 (31.2)
50,001+	110 (12.0)
Employed during last 12 months*, *n* (%)	
No	354 (38.5)
Yes	555 (60.3)

*1.2% missing;

**7% missing.

**Table 2.** Body mass index (BMI) status and waist circumference (WC) stratified by sex and total cholesterol (TC), low-density lipoprotein (LDL), and high-density lipoprotein (HDL) levels in millimoles per liter (mmol/L) (*n* = 920).

	BMI				WC		
	Normal weight*	Overweight	Obese	p-value**	Normal	Higher	p-value**
**Women (*n* = 479)**	98 (20.5)	143 (29.9)	238 (49.7)		349 (72.9%)	130 (27.1%)	
**TC**							
Lower <5.18	67 (68.4)	91 (63.6)	144 (60.5)	0.392	224 (64.2%)	78 (60.0%)	0.399
Higher ≥5.18	31 (31.6)	52 (36.4)	94 (39.5)		125 (35.8%)	52 (40.0%)	
**LDL**							
Lower <3.4	75 (76.5)	101 (70.6)	168 (70.6)	0.508	258 (73.9%)	86 (66.2%)	0.093
Higher ≥3.4	23 (23.5)	42 (29.4)	70 (29.4)		91 (26.1%)	44 (33.8%)	
**HDL**							
Lower <1.3	20 (20.4)	48 (33.6)	106 (44.5)	**<0.001**	105 (30.1%)	69 (53.1%)	**<0.001**
Higher ≥1.3	78 (79.6)	95 (66.4)	132 (55.5)		244 (69.9%)	61 (46.9%)	
**Men (*n* = 441)**	112 (25.4)	176 (39.9)	153 (34.7)		322 (73.0%)	119 (27.0%)	
**TC**							
Lower <5.18	80 (71.4)	89 (50.6)	82 (53.6)	**0.001**	184 (57.1%)	67 (56.3%)	0.874
Higher ≥5.18	32 (28.6)	87 (49.4)	71 (46.4)		138 (42.9%)	52 (43.7%)	
**LDL**							
Lower <3.4	82 (73.2)	95 (54.0)	81 (52.9)	**0.001**	191 (59.3%)	67 (56.3%)	0.569
Higher ≥3.4	30 (26.8)	81 (46.0)	72 (47.1)		131 (40.7%)	52 (43.7%)	
**HDL**							
Lower <1	16 (14.3)	41 (23.3)	42 (27.5)	**0.038**	61 (18.9%)	38 (31.9%)	**0.004**
Higher ≥1	96 (85.7)	135 (76.7)	111 (72.5)		261 (81.1%)	81 (68.1%)	

Results are represented as *n* (%);

*Seven women and nine men were underweight, with minimum BMIs of 16.84 and 15.75, respectively.

**p-value from Chi-square test. Bold values indicate statistically significant results.

**Table 3.** Gender-stratified association between BMI and total cholesterol (TC) levels, low-density lipoprotein (LDL), and high-density lipoprotein (HDL) levels.

	BMI status	BMI (categorical)		BMI (continuous)	
		Unadjusted OR^1^ [95% CI]	Adjusted^3^ aOR^2^ [95% CI]	Unadjusted *β* [95% CI]	Adjusted^3^ *β* [95% CI]
**Women**					
TC (continuous)				0.11 [−0.01 to 0.24]	0.04 [−0.11 to 0.19]
Higher TC	Normal	Reference	Reference		
	Overweight	1.24 [0.72–2.13]	0.95 [0.51–1.77]		
	Obese	1.41 [0.86–2.32]	1.06 [0.58–1.93]		
LDL (continuous)				0.12 [0.01–0.23]	0.10 [−0.03 to 0.23]
Higher LDL	Normal	Reference	Reference		
	Overweight	1.36 [0.75–2.45]	0.99 [0.51–1.94]		
	Obese	1.36 [0.79–2.34]	0.98 [0.51–1.88]		
HDL (continuous)				**−0.13 [−0.17 to −0.08]**	**−0.13 [−0.19 to −0.08]**
Lower HDL	Normal	Reference	Reference		
	Overweight	**1.97 [1.08–3.60]**	**2.23 [1.14–4.36]**		
	Obese	**3.13 [1.80–5.45]**	**3.38 [1.76–6.49]**		
**Men**					
TC (continuous)				0.07 [0.00–0.14]	0.13 [−0.04 to 0.29]
Higher TC	Normal	Reference	Reference		
	Overweight	**2.44 [1.47–4.05]**	**2.59 [1.49–4.50]**		
	Obese	**2.16 [1.29–3.64]**	**2.27 [1.28–4.02]**		
LDL (continuous)				0.05 [−0.01 to 0.12]	0.13 [−0.02 to 0.27]
Higher LDL	Normal	Reference	Reference		
	Overweight	**2.33 [1.40–3.89]**	2.59 [1.48–4.54]		
	Obese	**2.43 [1.44–4.11]**	**2.76 [1.55–4.93]**		
HDL (continuous)				**−0.05 [−0.06 to −0.03]**	**−0.08 [−0.12 to −0.04]**
Lower HDL	Normal	Reference	Reference		
	Overweight	**1.82 [0.97–3.44]**	**2.33 [1.16–4.70]**		
	Obese	**2.27 [1.20–4.29]**	**2.81 [1.39–5.71]**		

1 OR: odds ratio;

2 aOR: adjusted odds ratio;

3 adjusted for age (continuous), education, employment, income, ever smoked cigarette or water pipe, physically active (150+ minutes per week), history of diabetes, history of high blood pressure, and history of high cholesterol. Bold values indicate statistically significant results.

**Table 4.** Gender-stratified association between waist circumference (WC) and total cholesterol (TC) levels, low-density lipoprotein (LDL), and high-density lipoprotein (HDL) levels.

	WC (categorical)			WC (continuous)	
	WC levels	Unadjusted OR^1^ [95% CI]	Adjusted^3^ aOR^2^ [95% CI]	Unadjusted *β* [95% CI]	Adjusted^3^ *β* [95% CI]
**Women**					
TC (continuous)				0.06 [0.00–0.12]	0.04 [−0.11 to 0.19]
Higher TC	Normal	Reference	Reference		
	Higher	1.19 [0.79–1.81]	1.12 [0.66–1.91]		
LDL (continuous)				**0.07 [0.02–0.12]**	0.10 [−0.03 to 0.23]
Higher LDL	Normal	Reference	Reference		
	Higher	1.45 [0.94–2.24]	1.49 [0.86–2.58]]		
HDL (continuous)				**−0.08 [−0.10 to −0.06]**	**−0.13 [−0.19 to -0.08]**
Lower HDL	Normal	Reference	Reference		
	Higher	**2.63 [1.74–3.97]**	**2.35 [1.42–3.90]**		
**Men**					
TC (continuous)				0.05 [−0.01 to 0.12]	0.13 [−0.04 to 0.29]
Higher TC	Normal	Reference	Reference		
	Higher	1.03 [0.68–1.58]	0.87 [0.54–1.40]		
LDL (continuous)				0.04 [−0.01 to 0.10]	0.13 [−0.02 to 0.27]
Higher LDL	Normal	Reference	Reference		
	Higher	1.13 [0.74–1.73]]	1.08 [0.67–1.73]		
HDL (continuous)				**−0.04 [-0.06 to −0.03]**	**−0.08 [−0.12 to -0.04]**
Lower HDL	Normal	Reference	Reference		
	Higher	**2.01 [1.25–3.23]**	**2.12 [1.27–3.55]**		

1 OR: odds ratio;

2 aOR: adjusted odds ratio;

3 adjusted for age (continuous), education, employment, income, ever smoked cigarette or water pipe, physically active (150+ minutes per week), history of diabetes, history of high blood pressure, and history of high cholesterol. Bold values indicate statistically significant results.

**Supplementary Table 1.** Woman participants factors by total cholesterol (TC), low-density lipoprotein (LDL), and high-density lipoprotein (HDL) levels in millimoles per liter (mmol/L) (*n* = 920).

	TC				LDL			HDL		
	Total	Lower	Higher	p-value	Lower	Higher	value	Lower	Higher	value
N	479 (100.0)	302 (63.0)	177 (37.0)		344 (71.8)	135 (28.2)		174 (36.3)	305 (63.7)	
**Age, mean (SD)**	40.7 (13.1)	38.9 (13.5)	43.6 (11.8)	<0.001	39.3 (13.2)	44.1 (12.1)	<0.001	42.0 (13.0)	39.9 (13.1)	0.096
**Nationality**										
Non-Qatari	68 (14.2)	43 (14.2)	25 (14.1)	0.972	49 (14.2)	19 (14.1)	0.962	31 (17.8)	37 (12.1)	0.086
Qatar	411 (85.8)	259 (85.8)	152 (85.9)		295 (85.8)	116 (85.9)		143 (82.2)	268 (87.9)	
Highest level of education*, *n*(%)										
Primary of below	91 (19.1)	55 (18.3)	36 (20.5)	0.013	66 (19.3)	25 (18.7)	0.260	42 (24.3)	49 (16.2)	0.092
Secondary	216 (45.4)	151 (50.3)	65 (36.9)		162 (47.4)	54 (40.3)		75 (43.4)	141 (46.5)	
University+	169 (35.5)	94 (31.3)	75 (42.6)		114 (33.3)	55 (41.0)		56 (32.4)	113 (37.3)	
Own house*, *n*(%)										
No	119 (25.0)	72 (24.0)	47 (26.7)	0.511	84 (24.6)	35 (26.1)	0.724	50 (28.9)	69 (22.8)	0.137
Yes	357 (75.0)	228 (76.0)	129 (73.3)		258 (75.4)	99 (73.9)		123 (71.1)	234 (77.2)	
Total monthly income per month in QR**, *n*(%)										
<10,000	167 (37.8)	108 (38.8)	59 (36.0)	0.663	124 (39.1)	43 (34.4)	0.806	71 (45.2)	96 (33.7)	0.075
10,000-20,000	132 (29.9)	86 (30.9)	46 (28.0)		94 (29.7)	38 (30.4)		37 (23.6)	95 (33.3)	
20,001-50,000	117 (26.5)	69 (24.8)	48 (29.3)		81 (25.6)	36 (28.8)		40 (25.5)	77 (27.0)	
50,001+	26 (5.9)	15 (5.4)	11 (6.7)		18 (5.7)	8 (6.4)		9 (5.7)	17 (6.0)	
Employed During last 12 months*, *n*(%)										
No	270 (56.7)	173 (57.7)	97 (55.1)	0.587	191 (55.8)	79 (59.0)	0.538	110 (63.6)	160 (52.8)	0.022
Yes	206 (43.3)	127 (42.3)	79 (44.9)		151 (44.2)	55 (41.0)		63 (36.4)	143 (47.2)	
**Active (150+ minutes per week), *n*(%)**										
No	288 (60.1)	177 (58.6)	111 (62.7)	0.376	201 (58.4)	87 (64.4)	0.226	119 (68.4)	169 (55.4)	0.005
Yes	191 (39.9)	125 (41.4)	66 (37.3)		143 (41.6)	48 (35.6)		55 (31.6)	136 (44.6)	
Hours of sleep in a 24-hour period*, *n*(%)										
<5	53 (11.1)	33 (11.0)	20 (11.4)	0.028	36 (10.5)	17 (12.7)	0.019	27 (15.6)	26 (8.6)	0.038
5-8	373 (78.4)	244 (81.3)	129 (73.3)		278 (81.3)	95 (70.9)		132 (76.3)	241 (79.5)	
8+	50 (10.5)	23 (7.7)	27 (15.3)		28 (8.2)	22 (16.4)		14 (8.1)	36 (11.9)	
Ever smoked*, *n*(%)										
No	435 (91.4)	270 (90.0)	165 (93.8)	0.159	312 (91.2)	123 (91.8)	0.844	160 (92.5)	275 (90.8)	0.518
Yes	41 (8.6)	30 (10.0)	11 (6.2)		30 (8.8)	11 (8.2)		13 (7.5)	28 (9.2)	
Ever smoked water pipe (shisha)*, *n*(%)										
No	439 (92.2)	271 (90.3)	168 (95.5)	0.044	312 (91.2)	127 (94.8)	0.194	160 (92.5)	279 (92.1)	0.873
Yes	37 (7.8)	29 (9.7)	8 (4.5)		30 (8.8)	7 (5.2)		13 (7.5)	24 (7.9)	
Frequency eating fast food in a week*, *n*(%)										
Twice or less	248 (52.4)	155 (52.0)	93 (53.1)	0.248	175 (51.5)	73 (54.9)	0.281	83 (48.5)	165 (54.6)	0.436
3-7 times	93 (19.7)	65 (21.8)	28 (16.0)		73 (21.5)	20 (15.0)		37 (21.6)	56 (18.5)	
Never or rarely	132 (27.9)	78 (26.2)	54 (30.9)		92 (27.1)	40 (30.1)		51 (29.8)	81 (26.8)	
**Frequency eating red meat, *n*(%)**										
Never or rarely	233 (48.6)	155 (51.3)	78 (44.1)	0.130	175 (50.9)	58 (43.0)	0.096	75 (43.1)	158 (51.8)	0.173
1-3 times per week	187 (39.0)	116 (38.4)	71 (40.1)		133 (38.7)	54 (40.0)		74 (42.5)	113 (37.0)	
4-6 times per week	59 (12.3)	31 (10.3)	28 (15.8)		36 (10.5)	23 (17.0)		25 (14.4)	34 (11.1)	
**History of diabetes, *n*(%)**										
No	410 (85.6)	254 (84.1)	156 (88.1)	0.225	295 (85.8)	115 (85.2)	0.873	140 (80.5)	270 (88.5)	0.016
Yes	69 (14.4)	48 (15.9)	21 (11.9)		49 (14.2)	20 (14.8)		34 (19.5)	35 (11.5)	
**History of high cholesterol, *n*(%)**										
No	356 (74.3)	243 (80.5)	113 (63.8)	<0.001	271 (78.8)	85 (63.0)	<0.001	123 (70.7)	233 (76.4)	0.169
Yes	123 (25.7)	59 (19.5)	64 (36.2)		73 (21.2)	50 (37.0)		51 (29.3)	72 (23.6)	
**History of high blood pressure, *n*(%)**										
No	432 (90.2)	267 (88.4)	165 (93.2)	0.088	306 (89.0)	126 (93.3)	0.147	155 (89.1)	277 (90.8)	0.538
Yes	47 (9.8)	35 (11.6)	12 (6.8)		38 (11.0)	9 (6.7)		19 (10.9)	28 (9.2)	
**Father history: High blood pressure, *n*(%)**										
No	309 (64.5)	202 (66.9)	107 (60.5)	0.155	224 (65.1)	85 (63.0)	0.658	113 (64.9)	196 (64.3)	0.881
Yes	170 (35.5)	100 (33.1)	70 (39.5)		120 (34.9)	50 (37.0)		61 (35.1)	109 (35.7)	
**Father history: Heart disease, *n*(%)**										
No	396 (82.7)	248 (82.1)	148 (83.6)	0.676	288 (83.7)	108 (80.0)	0.333	144 (82.8)	252 (82.6)	0.970
Yes	83 (17.3)	54 (17.9)	29 (16.4)		56 (16.3)	27 (20.0)		30 (17.2)	53 (17.4)	
**Father history: Stroke, *n*(%)**										
No	454 (94.8)	290 (96.0)	164 (92.7)	0.109	327 (95.1)	127 (94.1)	0.663	166 (95.4)	288 (94.4)	0.644
Yes	25 (5.2)	12 (4.0)	13 (7.3)		17 (4.9)	8 (5.9)		8 (4.6)	17 (5.6)	
**Father history: Diabetes, *n*(%)**										
No	295 (61.6)	182 (60.3)	113 (63.8)	0.437	208 (60.5)	87 (64.4)	0.420	106 (60.9)	189 (62.0)	0.821
Yes	184 (38.4)	120 (39.7)	64 (36.2)		136 (39.5)	48 (35.6)		68 (39.1)	116 (38.0)	
**Father history: Overweight/obesity, *n*(%)**										
No	469 (97.9)	295 (97.7)	174 (98.3)	0.645	336 (97.7)	133 (98.5)	0.561	170 (97.7)	299 (98.0)	0.807
Yes	10 (2.1)	7 (2.3)	3 (1.7)		8 (2.3)	2 (1.5)		4 (2.3)	6 (2.0)	
**Mother history: High blood pressure, *n*(%)**										
No	275 (57.4)	173 (57.3)	102 (57.6)	0.942	199 (57.8)	76 (56.3)	0.757	92 (52.9)	183 (60.0)	0.129
Yes	204 (42.6)	129 (42.7)	75 (42.4)		145 (42.2)	59 (43.7)		82 (47.1)	122 (40.0)	
**Mother history: Heart disease, *n*(%)**										
No	412 (86.0)	269 (89.1)	143 (80.8)	0.012	297 (86.3)	115 (85.2)	0.744	148 (85.1)	264 (86.6)	0.649
Yes	67 (14.0)	33 (10.9)	34 (19.2)		47 (13.7)	20 (14.8)		26 (14.9)	41 (13.4)	
**Mother history: Stroke, *n*(%)**										
No	458 (95.6)	290 (96.0)	168 (94.9)	0.566	330 (95.9)	128 (94.8)	0.592	165 (94.8)	293 (96.1)	0.524
Yes	21 (4.4)	12 (4.0)	9 (5.1)		14 (4.1)	7 (5.2)		9 (5.2)	12 (3.9)	
**Mother history: Diabetes, *n*(%)**										
No	274 (57.2)	177 (58.6)	97 (54.8)	0.416	205 (59.6)	69 (51.1)	0.091	94 (54.0)	180 (59.0)	0.288
Yes	205 (42.8)	125 (41.4)	80 (45.2)		139 (40.4)	66 (48.9)		80 (46.0)	125 (41.0)	
**Mother history: Overweight/obesity, *n*(%)**										
No	453 (94.6)	292 (96.7)	161 (91.0)	0.008	328 (95.3)	125 (92.6)	0.231	164 (94.3)	289 (94.8)	0.816
Yes	26 (5.4)	10 (3.3)	16 (9.0)		16 (4.7)	10 (7.4)		10 (5.7)	16 (5.2)	
**Treatment for cholesterol, *n*(%)**										
No	388 (81.0)	252 (83.4)	136 (76.8)	0.075	285 (82.8)	103 (76.3)	0.100	131 (75.3)	257 (84.3)	0.016
Yes	91 (19.0)	50 (16.6)	41 (23.2)		59 (17.2)	32 (23.7)		43 (24.7)	48 (15.7)	

* *p*-values from Pearson chi-square tests and t-tests ; ^*^<1.25% ;

** 7.72% missing.

**Supplementary Table 2.** Men participants factors by total cholesterol (TC), low-density lipoprotein (LDL), and high-density lipoprotein (HDL) levels in millimoles per liter (mmol/L) (*n* = 920).

	TC				LDL			HDL		
	Total	Lower	Higher	p-value	Lower	Higher	p-value	Lower	Higher	*p*-value
N	441 (100.0)	251 (56.9)	190 (43.1)		258 (58.5)	183 (41.5)		99 (22.4)	342 (77.6)	
**Age, mean (SD)**	39.8 (13.1)	38.9 (14.1)	41.1 (11.6)	0.079	39.4 (14.0)	40.4 (11.8)	0.447	41.2 (12.9)	39.5 (13.2)	0.252
**Nationality, *n*(%)**										
Non-Qatari	107 (24.3)	61 (24.3)	46 (24.2)	0.982	64 (24.8)	43 (23.5)	0.752	23 (23.2)	84 (24.6)	0.786
Qatar	334 (75.7)	190 (75.7)	144 (75.8)		194 (75.2)	140 (76.5)		76 (76.8)	258 (75.4)	
Highest level of education*, *n*(%)										
Primary of below	31 (7.2)	19 (7.7)	12 (6.4)	0.341	22 (8.7)	9 (5.0)	0.146	7 (7.1)	24 (7.2)	0.952
Secondary	204 (47.1)	122 (49.6)	82 (43.9)		123 (48.8)	81 (44.8)		48 (48.5)	156 (46.7)	
University+	198 (45.7)	105 (42.7)	93 (49.7)		107 (42.5)	91 (50.3)		44 (44.4)	154 (46.1)	
Own house*, *n*(%)										
No	159 (36.7)	94 (38.2)	65 (34.8)	0.460	97 (38.5)	62 (34.3)	0.367	45 (45.5)	114 (34.1)	0.040
Yes	274 (63.3)	152 (61.8)	122 (65.2)		155 (61.5)	119 (65.7)		54 (54.5)	220 (65.9)	
Total monthly income per month in QR**, *n*(%)										
<10,000	58 (14.0)	43 (18.4)	15 (8.3)	0.012	39 (16.2)	19 (10.9)	0.048	15 (15.8)	43 (13.5)	0.772
10,000-20,000	102 (24.6)	59 (25.2)	43 (23.9)		67 (27.9)	35 (20.1)		20 (21.1)	82 (25.7)	
20,001-50,000	170 (41.1)	84 (35.9)	86 (47.8)		87 (36.2)	83 (47.7)		39 (41.1)	131 (41.1)	
50,001+	84 (20.3)	48 (20.5)	36 (20.0)		47 (19.6)	37 (21.3)		21 (22.1)	63 (19.7)	
Employed During last 12 months*, *n*(%)										
No	84 (19.4)	54 (22.0)	30 (16.0)	0.124	53 (21.0)	31 (17.1)	0.311	23 (23.2)	61 (18.3)	0.272
Yes	349 (80.6)	192 (78.0)	157 (84.0)		199 (79.0)	150 (82.9)		76 (76.8)	273 (81.7)	
**Active (150+ minutes per week), *n*(%)**										
No	194 (44.0)	106 (42.2)	88 (46.3)	0.392	115 (44.6)	79 (43.2)	0.770	46 (46.5)	148 (43.3)	0.573
Yes	247 (56.0)	145 (57.8)	102 (53.7)		143 (55.4)	104 (56.8)		53 (53.5)	194 (56.7)	
Hours of sleep in a 24-hour period*, *n*(%)										
<5	55 (12.7)	31 (12.6)	24 (12.8)	0.927	31 (12.3)	24 (13.3)	0.401	8 (8.1)	47 (14.1)	0.227
5-8	324 (74.8)	183 (74.4)	141 (75.4)		185 (73.4)	139 (76.8)		76 (76.8)	248 (74.3)	
8+	54 (12.5)	32 (13.0)	22 (11.8)		36 (14.3)	18 (9.9)		15 (15.2)	39 (11.7)	
Ever smoked*, *n*(%)										
No	152 (35.1)	95 (38.6)	57 (30.5)	0.079	98 (38.9)	54 (29.8)	0.052	31 (31.3)	121 (36.2)	0.368
Yes	281 (64.9)	151 (61.4)	130 (69.5)		154 (61.1)	127 (70.2)		68 (68.7)	213 (63.8)	
Ever smoked water pipe (shisha)*, *n*(%)										
No	199 (46.0)	118 (48.0)	81 (43.3)	0.336	125 (49.6)	74 (40.9)	0.073	42 (42.4)	157 (47.0)	0.422
Yes	234 (54.0)	128 (52.0)	106 (56.7)		127 (50.4)	107 (59.1)		57 (57.6)	177 (53.0)	
Frequency eating fast food in a week***, *n*(%)										
Twice or less	231 (54.0)	122 (50.2)	109 (58.9)	0.169	125 (50.2)	106 (59.2)	0.181	54 (55.1)	177 (53.6)	0.311
3-7 times	96 (22.4)	61 (25.1)	35 (18.9)		60 (24.1)	36 (20.1)		17 (17.3)	79 (23.9)	
Never or rarely	101 (23.6)	60 (24.7)	41 (22.2)		64 (25.7)	37 (20.7)		27 (27.6)	74 (22.4)	
**Frequency eating red meat, *n*(%)**										
Never or rarely	134 (30.4)	82 (32.7)	52 (27.4)	0.449	86 (33.3)	48 (26.2)	0.261	21 (21.2)	113 (33.0)	0.072
1-3 times per week	183 (41.5)	99 (39.4)	84 (44.2)		101 (39.1)	82 (44.8)		48 (48.5)	135 (39.5)	
4-6 times per week	124 (28.1)	70 (27.9)	54 (28.4)		71 (27.5)	53 (29.0)		30 (30.3)	94 (27.5)	
**History of diabetes, *n*(%)**										
No	375 (85.0)	209 (83.3)	166 (87.4)	0.232	214 (82.9)	161 (88.0)	0.144	80 (80.8)	295 (86.3)	0.181
Yes	66 (15.0)	42 (16.7)	24 (12.6)		44 (17.1)	22 (12.0)		19 (19.2)	47 (13.7)	
**History of high cholesterol, *n*(%)**										
No	322 (73.0)	195 (77.7)	127 (66.8)	0.011	199 (77.1)	123 (67.2)	0.021	70 (70.7)	252 (73.7)	0.557
Yes	119 (27.0)	56 (22.3)	63 (33.2)		59 (22.9)	60 (32.8)		29 (29.3)	90 (26.3)	
**History of high blood pressure, *n*(%)**										
No	373 (84.6)	214 (85.3)	159 (83.7)	0.650	215 (83.3)	158 (86.3)	0.389	85 (85.9)	288 (84.2)	0.689
Yes	68 (15.4)	37 (14.7)	31 (16.3)		43 (16.7)	25 (13.7)		14 (14.1)	54 (15.8)	
**Father history: High blood pressure, *n*(%)**										
No	258 (58.5)	148 (59.0)	110 (57.9)	0.821	151 (58.5)	107 (58.5)	0.990	55 (55.6)	203 (59.4)	0.499
Yes	183 (41.5)	103 (41.0)	80 (42.1)		107 (41.5)	76 (41.5)		44 (44.4)	139 (40.6)	
**Father history: Heart disease, *n*(%)**										
No	355 (80.5)	201 (80.1)	154 (81.1)	0.798	203 (78.7)	152 (83.1)	0.253	81 (81.8)	274 (80.1)	0.707
Yes	86 (19.5)	50 (19.9)	36 (18.9)		55 (21.3)	31 (16.9)		18 (18.2)	68 (19.9)	
**Father history: Stroke, *n*(%)**										
No	411 (93.2)	238 (94.8)	173 (91.1)	0.120	244 (94.6)	167 (91.3)	0.173	94 (94.9)	317 (92.7)	0.432
Yes	30 (6.8)	13 (5.2)	17 (8.9)		14 (5.4)	16 (8.7)		5 (5.1)	25 (7.3)	
**Father history: Diabetes, *n*(%)**										
No	260 (59.0)	149 (59.4)	111 (58.4)	0.842	150 (58.1)	110 (60.1)	0.679	57 (57.6)	203 (59.4)	0.751
Yes	181 (41.0)	102 (40.6)	79 (41.6)		108 (41.9)	73 (39.9)		42 (42.4)	139 (40.6)	
**Father history: Overweight/obesity, *n*(%)**										
No	415 (94.1)	235 (93.6)	180 (94.7)	0.624	244 (94.6)	171 (93.4)	0.619	94 (94.9)	321 (93.9)	0.685
Yes	26 (5.9)	16 (6.4)	10 (5.3)		14 (5.4)	12 (6.6)		5 (5.1)	21 (6.1)	
**Mother history: High blood pressure, *n*(%)**										
No	257 (58.3)	150 (59.8)	107 (56.3)	0.467	154 (59.7)	103 (56.3)	0.475	56 (56.6)	201 (58.8)	0.695
Yes	184 (41.7)	101 (40.2)	83 (43.7)		104 (40.3)	80 (43.7)		43 (43.4)	141 (41.2)	
**Mother history: Heart disease, *n*(%)**										
No	388 (88.0)	220 (87.6)	168 (88.4)	0.805	227 (88.0)	161 (88.0)	0.998	82 (82.8)	306 (89.5)	0.073
Yes	53 (12.0)	31 (12.4)	22 (11.6)		31 (12.0)	22 (12.0)		17 (17.2)	36 (10.5)	
**Mother history: Stroke, *n*(%)**										
No	424 (96.1)	243 (96.8)	181 (95.3)	0.403	249 (96.5)	175 (95.6)	0.635	95 (96.0)	329 (96.2)	0.913
Yes	17 (3.9)	8 (3.2)	9 (4.7)		9 (3.5)	8 (4.4)		4 (4.0)	13 (3.8)	
**Mother history: Diabetes, *n*(%)**										
No	256 (58.0)	152 (60.6)	104 (54.7)	0.220	156 (60.5)	100 (54.6)	0.222	54 (54.5)	202 (59.1)	0.422
Yes	185 (42.0)	99 (39.4)	86 (45.3)		102 (39.5)	83 (45.4)		45 (45.5)	140 (40.9)	
**Mother history: Overweight/obesity, *n*(%)**										
No	399 (90.5)	229 (91.2)	170 (89.5)	0.533	233 (90.3)	166 (90.7)	0.888	89 (89.9)	310 (90.6)	0.824
Yes	42 (9.5)	22 (8.8)	20 (10.5)		25 (9.7)	17 (9.3)		10 (10.1)	32 (9.4)	
**Treatment for cholesterol, *n*(%)**										
No	366 (83.0)	204 (81.3)	162 (85.3)	0.270	210 (81.4)	156 (85.2)	0.289	84 (84.8)	282 (82.5)	0.577
Yes	75 (17.0)	47 (18.7)	28 (14.7)		48 (18.6)	27 (14.8)		15 (15.2)	60 (17.5)	

* *p*-values from Pearson chi-square tests and t-tests ; *<2% ;

** 6% missing;

*** 2.95% missing.

**Table:** STROBE STATEMENT

	Item No	Recommendation	Page
**Title and abstract**	1	(a) Indicate the study’s design with a commonly used term in the title or the abstract	1
		(b) Provide in the abstract an informative and balanced summary of what was done and what was found	3,4
**Introduction**			
Background/ rationale	2	Explain the scientific background and rationale for the investigation being reported	5,6
Objectives	3	State specific objectives, including any prespecified hypotheses	6
**Methods**			
Study design	4	Present key elements of study design early in the paper	7
Setting	5	Describe the setting, locations, and relevant dates, including periods of recruitment, exposure, follow-up, and data collection	7
Participants	6	(a) Give the eligibility criteria, and the sources and methods of selection of participants	7
Variables	7	Clearly define all outcomes, exposures, predictors, potential confounders, and effect modifiers. Give diagnostic criteria, if applicable	7,8
Data sources/ measurement	8*	For each variable of interest, give sources of data and details of methods of assessment (measurement). Describe comparability of assessment methods if there is more than one group	7,8
Bias	9	Describe any efforts to address potential sources of bias	NA
Study size	10	Explain how the study size was arrived at	9
Quantitative variables	11	Explain how quantitative variables were handled in the analyses. If applicable, describe which groupings were chosen and why	7,8
Statistical methods	12	(a) Describe all statistical methods, including those used to control for confounding	8
		(b) Describe any methods used to examine subgroups and interactions	NA
		(c) Explain how missing data were addressed	7
		(d) If applicable, describe analytical methods taking account of sampling strategy	NA
		(e) Describe any sensitivity analyses	NA
**Results**			
Participants	13*	(a) Report numbers of individuals at each stage of study—eg numbers potentially eligible, examined for eligibility, confirmed eligible, included in the study, completing follow-up, and analyzed	9
		(b) Give reasons for non-participation at each stage	NA
		(c) Consider use of a flow diagram	NA
Descriptive data	14*	(a) Give characteristics of study participants (eg demographic, clinical, social) and information on exposures and potential confounders	9,21
		(b) Indicate number of participants with missing data for each variable of interest	21,26-31
Outcome data	15*	Report numbers of outcome events or summary measures	9,22
Main results	16	(a) Give unadjusted estimates and, if applicable, confounder-adjusted estimates and their precision (eg, 95% confidence interval). Make clear which confounders were adjusted for and why they were included	11, 25,26
		(b) Report category boundaries when continuous variables were categorized	7,8
		(c) If relevant, consider translating estimates of relative risk into absolute risk for a meaningful time period	NA
Other analyses	17	Report other analyses done—eg analyses of subgroups and interactions, and sensitivity analyses	NA
**Discussion**			
Key results	18	Summarise key results with reference to study objectives	13
Limitations	19	Discuss limitations of the study, taking into account sources of potential bias or imprecision. Discuss both direction and magnitude of any potential bias	15,16
Interpretation	20	Give a cautious overall interpretation of results considering objectives, limitations, multiplicity of analyses, results from similar studies, and other relevant evidence	13,14,15
Generalisability	21	Discuss the generalisability (external validity) of the study results	15,16
**Other information**			
Funding	22	Give the source of funding and the role of the funders for the present study and, if applicable, for the original study on which the present article is based	18
